# Peritrophic matrix-degrading proteins are dispensable virulence factors in a virulent *Melissococcus plutonius* strain

**DOI:** 10.1038/s41598-021-88302-8

**Published:** 2021-04-22

**Authors:** Keiko Nakamura, Kayo Okumura, Mariko Harada, Mariko Okamoto, Masatoshi Okura, Daisuke Takamatsu

**Affiliations:** 1 Research and Business Promotion Division, Research Institute for Animal Science in Biochemistry and Toxicology, Sagamihara, Kanagawa 252-0132 Japan; 2 Department of Veterinary Medicine, Obihiro University of Agriculture and Veterinary Medicine, Obihiro, Hokkaido 080-8555 Japan; 3 Division of Bacterial and Parasitic Disease, National Institute of Animal Health, National Agriculture and Food Research Organization, Tsukuba, Ibaraki 305-0856 Japan; 4 The United Graduate School of Veterinary Sciences, Gifu University, Gifu, Gifu 501-1193 Japan

**Keywords:** Microbiology, Bacteria, Bacterial pathogenesis, Entomology

## Abstract

European foulbrood (EFB) caused by *Melissococcus plutonius* is a major bacterial disease of honey bees. Strains of the causative agent exhibit genetic heterogeneity, and the degree of virulence varies among strains. In bee larvae orally infected with the highly virulent strains, ingested bacterial cells colonize the larval midgut and proliferate within the sac of the peritrophic matrix (PM), a barrier lining the midgut epithelium. However, the barrier is degraded during the course of infection, and *M. plutonius* cells eventually directly interact with the midgut epithelium. As *M. plutonius* possesses genes encoding putative PM-degrading proteins (enhancin, a chitin-binding domain-containing protein and endo-α-*N*-acetylgalactosaminidase), we constructed PM-degrading protein gene-knockout mutants from a highly virulent *M. plutonius* strain and investigated their role in the pathogenesis of EFB. In larvae infected with the triple-knockout mutant, which has no PM-degrading protein genes, *M. plutonius* that proliferated in the larval midguts was confined to the sac of the PM. However, the midgut epithelial cells degenerated over time, and the mutant killed approximately 70–80% of bee brood, suggesting that although the PM-degrading proteins are involved in the penetration of the PM by *M. plutonius*, they are not indispensable virulence factors in the highly virulent *M. plutonius* strain.

## Introduction

As the western honey bee (*Apis mellifera* L.) is the most important commercial pollinator aiding in the production of a variety of agricultural and horticultural crops, diseases of the western honey bees have a great economic impact worldwide. European foulbrood (EFB) caused by a Gram-positive lanceolate coccus *Melissococcus plutonius*^1^ is one of the globally distributed and economically important infectious diseases of honey bees^2,3^. EFB affects mainly young larvae and usually kills them 1–2 days before being sealed in their cells^3,4^. However, the degree of virulence varies among *M. plutonius* strains, presumably among genotypes. *M. plutonius* strains can be grouped into three clonal complexes (CC3, CC12 and CC13) by the multilocus sequence typing analysis (https://pubmlst.org/mplutonius/)^5–8^. In previous in vitro experimental infections of *A. mellifera* larvae^9^, the representative CC12 strain (DAT561) was extremely virulent and killed all bees before pupation, whereas the representative CC3 strain (DAT606) was less virulent than the CC12 strain, and a part of the infected larvae pupated. In contrast, the representative CC13 strain (DAT585) had negligible effects on the mortality of bee larvae^9^. Lewkowski and Erler^10^ also showed virulence differences among *M. plutonius* strains with different genetic backgrounds. In their study, a European CC3 strain (49.3 from Switzerland) had consistently higher virulence against honey bee larvae than European CC13 strains (119 from Switzerland and 4–127 from Sweden). More recently, Grossar et al.^11^ quantified the virulence of 16 CC3 and CC13 isolates from five European countries using a standardized in vitro assay. Although the degree of virulence was independent of the sequence types of the tested *M. plutonius* isolates in the study, virulence varied greatly among the 16 isolates^11^. However, the genetic basis of the distinct pathological characteristics of different *M. plutonius* strains has not been fully elucidated.

In a recent study, Djukic et al.^12^ analyzed the genome sequences of 14 *M. plutonius* strains, and identified many putative virulence factors, which may be involved in the pathogenesis of EFB, including antimicrobial activity, tyramine production, glycoprotein degradation, host cell adhesion and cell envelope formation. Among the putative virulence factors, melissotoxin A, which shares 33% amino acid sequence identity with an epsilon toxin ETX/mosquitocidal toxin MTX2 family protein of *Brevibacillus laterosporus*, and a putative extracellular matrix binding protein were found to be encoded on a 19.4-kbp plasmid, pMP19^12^. We recently demonstrated that the highly virulent strain DAT561 (CC12) and moderately virulent strain DAT606 (CC3) possess pMP19, whereas the avirulent strain DAT585 (CC13) does not have the plasmid^13–16^. As the loss of pMP19 from CC3 strains resulted in a significant decrease in the virulence of the strains, pMP19 was considered to be a virulence determinant at least for CC3 strains^16^. However, irrespective of the presence of pMP19, the CC12 strain DAT561 was virulent and killed more than 80% of the honey bee brood in our in vitro infection model^16^, strongly suggesting the presence of unidentified pathomechanisms functioning in the virulence of CC12 strains.

In American foulbrood (AFB), another contagious bacterial infectious disease of honey bee brood, the infectious cycle begins when a larva eats spores of the causative agent *Paenibacillus larvae*. Ingested spores germinate in the larval midgut and vegetative *P. larvae* cells proliferate within the midgut^17,18^. In the larval midgut, the peritrophic matrix (PM), a chitin and glycoprotein layer that covers the lumen side of the midgut epithelium, protects the midgut epithelium from damage by pathogens^19^; however, *P. larvae* degrades this protective layer by the chitin-binding and -degrading protein *Pl*CBP49, breaches the gut epithelium, invades the hemocoel and proliferates in the paracellular space^17,18,20,21^. The infected larvae finally die from septicemia^22–24^. In the absence of *Pl*CBP49 expression, PM degradation was markedly reduced and *P. larvae* virulence was nearly abolished; therefore, the PM-degrading protein *Pl*CBP49 is considered a key virulence factor in *P. larvae*^21^.

*M. plutonius* is also known to colonize the larval midgut. However, the impact on the midgut differs depending on the strain ingested. When *A. mellifera* larvae were experimentally infected with the highly virulent strain DAT561, orally ingested bacterial cells were localized on the surface of the PM. The DAT561 cells then rapidly multiplied on the PM and eventually almost completely occupied the midgut lumen^25^. Similar to AFB, the PM was degraded during the course of DAT561 infection and *M. plutonius* directly interacted with the midgut epithelium^25^. In the infected larvae, degeneration of the epithelial cells was also observed^25^. In contrast, when *A. mellifera* larvae were infected with a low virulent strain of CC13 (LMG 20360, the type strain of *M. plutonius*), the intestinal wall was not destroyed, and the PM and intestinal epithelium remained intact during the 15-day experimental period^26^. Therefore, as with AFB by *P. larvae*, PM-degrading proteins may be indispensable in the pathogenesis of EFB by the highly virulent *M. plutonius* strain DAT561 of CC12.

In a recent comparative genome analysis of *M. plutonius* strains, Djukic et al.^12^ found three putative PM-degrading protein genes in the genomes of *M. plutonius*. One of the genes encodes the chitin-binding domain-containing protein (Cbp), which exhibited 37% amino acid sequence similarity to *Pl*CBP49 of *P. larvae* (Supplementary Fig. S1a). The other two genes encode enhancin (peptidase M60 family protein/enhancin family protein, Efp) and a putative endo-α-*N*-acetylgalactosaminidase (endo-α-GalNAc-ase)^12^. Enhancins, which belong to a class of metalloproteases originally found in some baculoviruses, are known to promote viral infection by degrading the PM of the insect midgut^27^, whereas endo-α-GalNAc-ase is the enzyme that catalyzes the hydrolysis of the *O*-glycosidic bond between α-GalNAc at the reducing end of mucin-type sugar chains (*O*-glycan) and serine/threonine residues of proteins^28^. Among the three putative PM-degrading proteins, the expression of Efp and endo-α-GalNAc-ase in naturally infected larvae has been confirmed^12^. However, it is unknown whether these putative PM-degrading proteins affect the integrity of the PM of honey bee larvae and function as key virulence factors of *M. plutonius*. As our research group recently determined the genome sequences of the highly virulent *M. plutonius* strain DAT561, moderately virulent strain DAT606, and avirulent strains DAT585 and ATCC 35311 (= LMG 20360, the type strain of *M. plutonius*)^14,15,29^, in order to investigate the role of the putative PM-degrading proteins in the pathogenesis of EFB, in particular that by the highly virulent strain DAT561, we compared amino acid sequences of the proteins among the strains. In addition, we constructed a series of putative PM-degrading protein gene-deficient mutants from DAT561 and investigated their characteristics using the in vitro* A. mellifera* larvae infection model.

## Results and discussion

### Comparison of the putative PM-degrading proteins among *M. plutonius* strains

**Figure 1:**
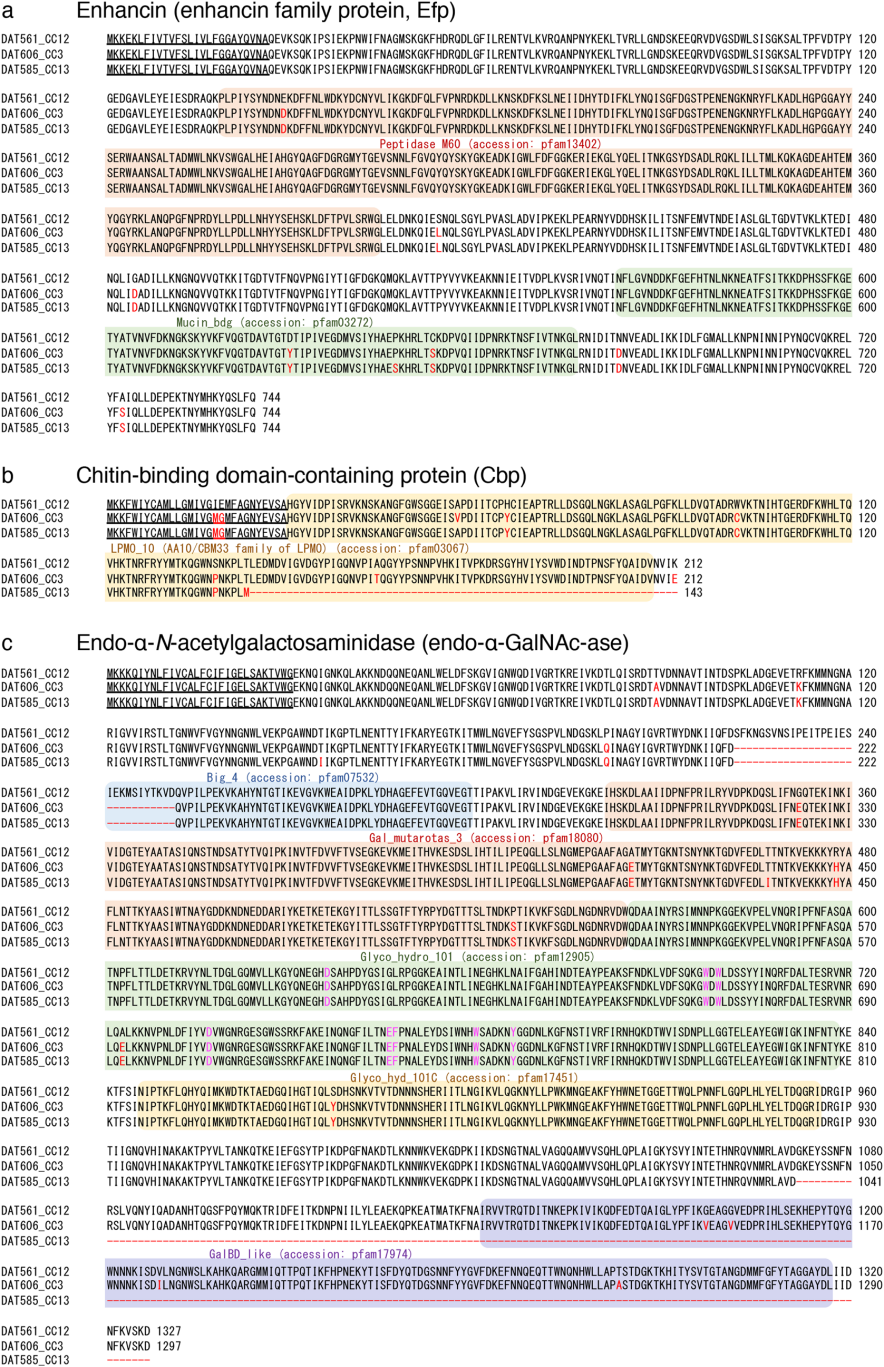
Multiple sequence alignment of enhancin (**a**), chitin-binding domain-containing protein (**b**) and endo-α-*N*-acetylgalactosaminidase (**c**) of *M. plutonius* DAT561 (CC12), DAT606 (CC3) and DAT585 (CC13). The multiple alignment was computed using ClustalW (https://clustalw.ddbj.nig.ac.jp/). Conserved domains are highlighted with colored backgrounds. Red letters represent amino acid residues different from those of DAT561. Hyphens indicate deleted/truncated regions in DAT606 and DAT585. Magenta letters in the endo-α-*N*-acetylgalactosaminidases are putative catalytic/active sites. Underlined sequences are putative signal peptides predicted by SignalP-5.0 (http://www.cbs.dtu.dk/services/SignalP/).

Efp of *M. plutoniu*s has a putative signal peptide and two conserved domains (Peptidase_M60 [pfam13402] and Mucin_bdg [putative mucin or carbohydrate-binding module; pfam03272]) (Fig. 1a). Peptidase_M60 family proteins in some baculoviruses and bacterial species contain a zinc metallopeptidase motif (HEXXHX(8,28)E) and possess mucinase activity (https://www.ncbi.nlm.nih.gov/Structure/cdd/cddsrv.cgi?uid=pfam13402), whereas Mucin_bdg is the putative binding domain for the substrates of enhancin and other similar metallopeptidases (https://www.ncbi.nlm.nih.gov/Structure/cdd/cddsrv.cgi?uid=pfam03272). In the previous genome analysis of *M. plutonius*^12^, Efp was found to be conserved in all European strains analyzed (Table 1). Consistent with the previous results, this protein was well conserved among the highly virulent (DAT561 of CC12), moderately virulent (DAT606 of CC3) and avirulent (DAT585 of CC13) strains in Japan (Fig. 1a and Table 1). Amino acid sequences of the proteins were more than 98.9% identical among the three strains.

**Table 1:** State of genes encoding putative PM-degrading proteins of *M. plutonius*.

Putative PM-degrading proteins (gene name)	State of the PM-degrading protein genes (protein id/accession no.)^a^				
	DAT561, CC12 strain	DAT606, CC3 strain	DAT585, CC13 strain	European CC3 strains^b^^12^	European CC13 strains^c^^12, 29^
Enhancin (*efp*)	Intact (BBC61709.1)	Intact (BBD17568.1)	Intact (BBD15944.1)	Intact	Intact
Chitin-binding domain-containing protein (*cbp*)	Intact (BBC61089.1)	Intact (BBD16660.1)	Pseudo^d^ (AP018524)	Intact	Intact
Endo-α-*N*-acetylgalactosaminidase (*eng*)	Intact (BBC60355.1)	Probably intact^e^ (BBD16245.1)	Pseudo^f^ (AP018524)	Probably intact^e^	Pseudo^f^

^a^CC, clonal complex.

^b^Strains 764-5B, 765-6B, 49.3, S1, 60, 21.1, B5, H6 and L9.

^c^Strains ATCC 35311 (LMG 20360), 119, 90.0 and 82.

^d^Chitin-binding domain-containing protein of DAT585 was truncated due to a 193-bp deletion.

^e^Endo-α-*N*-acetylgalactosaminidases of CC3 strains were 30 amino acids shorter than that of DAT561 due to a 90-bp deletion.

^f^Endo-α-*N*-acetylgalactosaminidases of CC13 strains were truncated due to nonsense mutations.

Cbp of *M. plutoniu*s possesses a putative signal peptide and the LPMO_10 domain (lytic polysaccharide mono-oxygenase, cellulose-degrading domain; pfam03067), which is also annotated as the module belonging to the auxiliary activities 10 (AA10) family or chitin binding module 33 (CBM33) family of lytic polysaccharide monooxygenases^21^ (Fig. 1b). Some AA10 family members, including CBP21 expressed by *Serratia marcescens* and *Ef*CBM33A expressed by *Enterococcus faecalis*, are capable of degrading crystalline chitin via a novel, copper-dependent, oxidative enzymatic mechanism^30–32^, and amino acid residues in the AA10 family members, which are involved in chitin binding and degradation, were well conserved in Cbp of *M. plutoniu*s (Supplementary Fig. S1b). The LPMO_10 domain is also conserved in *Pl*CBP49 of *P. larvae*^21^ (Supplementary Fig. S1a). However, compared with *Pl*CBP49 with 443 amino acids, Cbp of *M. plutoniu*s is short (212 amino acids in length) and does not have other domains conserved in *Pl*CBP49 (Supplementary Fig. S1a). The size of the *cbp* genes was the same (639 bp) between DAT561 of CC12 and DAT606 of CC3, and their amino acid sequences were 96.2% identical (Fig. 1b). European CC3 and CC13 strains also possess an intact *cbp* gene of 639 bp in their genomes (Table 1 and Supplementary Fig. S1c). However, in the *cbp* gene of DAT585, a 193-bp coding region was deleted, resulting in truncation of the C-terminal region of the protein (Fig. 1b and Supplementary Fig. S1c); therefore, this gene was considered to be a pseudogene in DAT585 (Table 1).

Endo-α-GalNAc-ase of *M. plutoniu*s DAT561 has a putative signal peptide and five conserved domains (Big_4 [bacterial Ig-like domain group 4 found in a variety of bacterial surface proteins; pfam07532], Gal_mutarotas_3 [galactose mutarotase-like fold domain; pfam18080], Glyco_hydro_101 [endo-alpha-*N*-acetylgalactosaminidase domain; pfam12905], Glyco_hyd_101C [glycosyl hydrolase 101 beta sandwich domain; pfam17451] and GalBD_like [galactose-binding domain-like domain; pfam17974]) (Fig. 1c). Among the five domains, Glyco_hydro_101 and Glyco_hyd_101C, which are putative enzymatic regions of endo-α-GalNAc-ase, and Gal_mutarotas_3, which is also found in endo-α-GalNAc-ase of *Streptococcus pneumoniae*, were well conserved in all *M. plutonius* strains investigated (Fig. 1c and Table 1). In contrast, a part of the Big_4 domain was lost in both Japanese and European CC3 and CC13 strains due to a 90-bp deletion in the endo-α-GalNAc-ase gene (*eng*). Moreover, in CC13 strains, the enzyme was truncated due to nonsense mutations at the C-terminal region, resulting in deletion of the GalBD_like domain (Fig. 1c and Table 1). The common function of the GalBD_like domain is binding specific ligands, such as cell-surface-attached carbohydrate substrates (https://www.ncbi.nlm.nih.gov/Structure/cdd/cddsrv.cgi?uid=pfam17974); therefore, endo-α-GalNAc-ase of CC13 strains may not be functional. Indeed, the *eng* gene of CC13 strains was annotated as pseudo in the GenBank database (Table 1)^12^.

In summary, two of the three putative PM-degrading protein genes (*cbp* and *eng*) were considered to be pseudogenes in the avirulent strain DAT585, and a 90-bp in-frame deletion was found in the *eng* gene in the moderately virulent strain DAT606. In contrast, in the highly virulent strain DAT561, all three genes were considered to be intact and they may therefore be involved in the high virulence of the CC12 strain. However, the true roles of these proteins in the pathogenesis of EFB cannot be determined by only in silico analyses. In addition to evaluation of the in silico data, it is essential to analyze the genes using isogenic mutants.

### Construction of the putative PM-degrading protein gene deletion mutants

**Figure 2:**
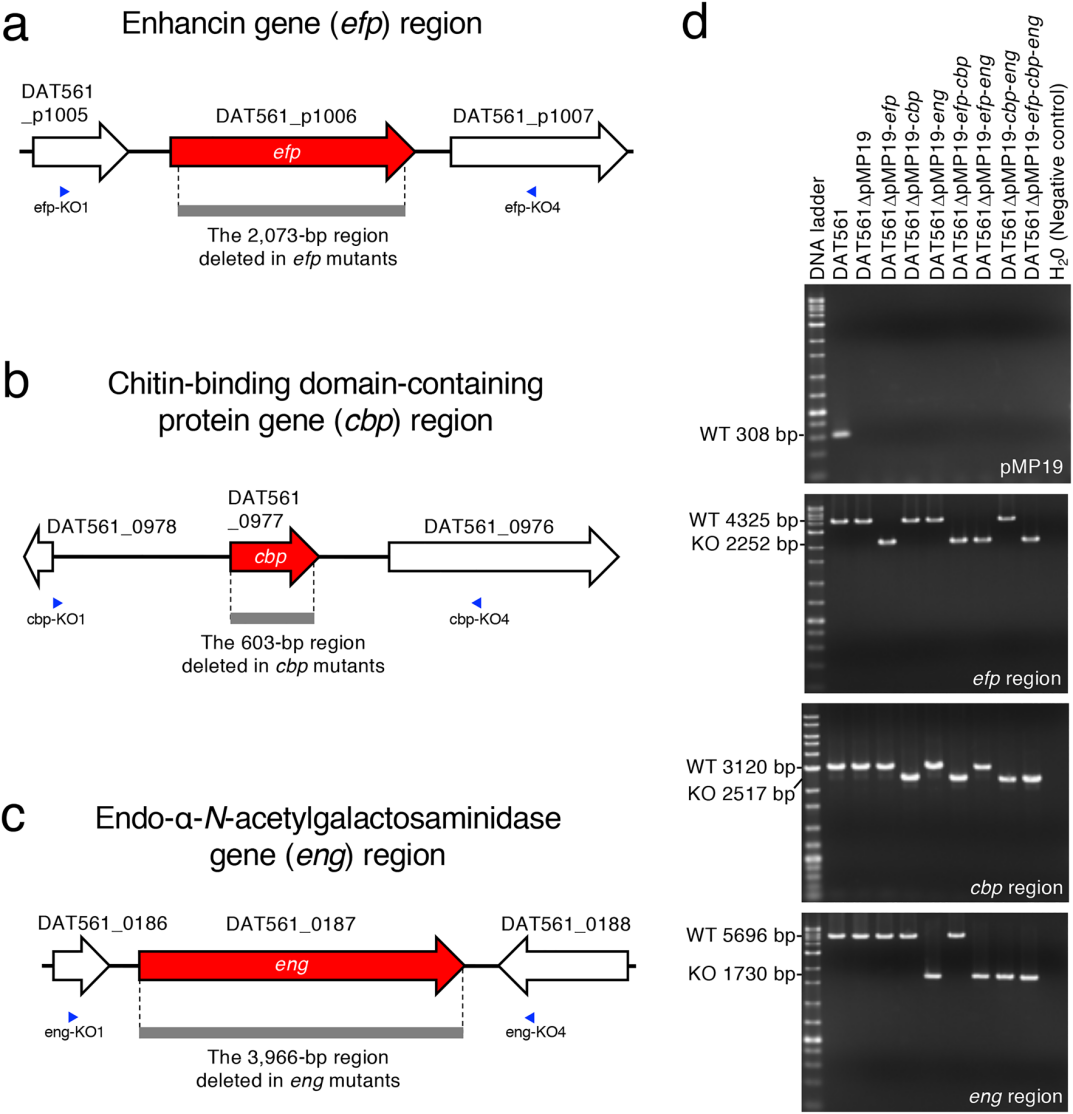
Schematic representation of the enhancin gene (*efp*) (**a**), chitin-binding domain-containing protein gene (*cbp*) (**b**) and endo-α-*N*-acetylgalactosaminidase gene (*eng*) (**c**) regions of DAT561. Gray bars represent regions deleted in the mutants constructed in this study. Blue arrowheads represent primers used to confirm deletion of the target genes. (**d**) Detection of pMP19 and confirmation of the target gene deletions in the constructed mutants by PCR. The primers, enzyme and PCR conditions are listed in Supplementary Table S1. Except for DAT561, *M. plutonius* strains used in this study lost pMP19. In each gene deletion mutant, the target regions were deleted as expected.

**Figure 3:**
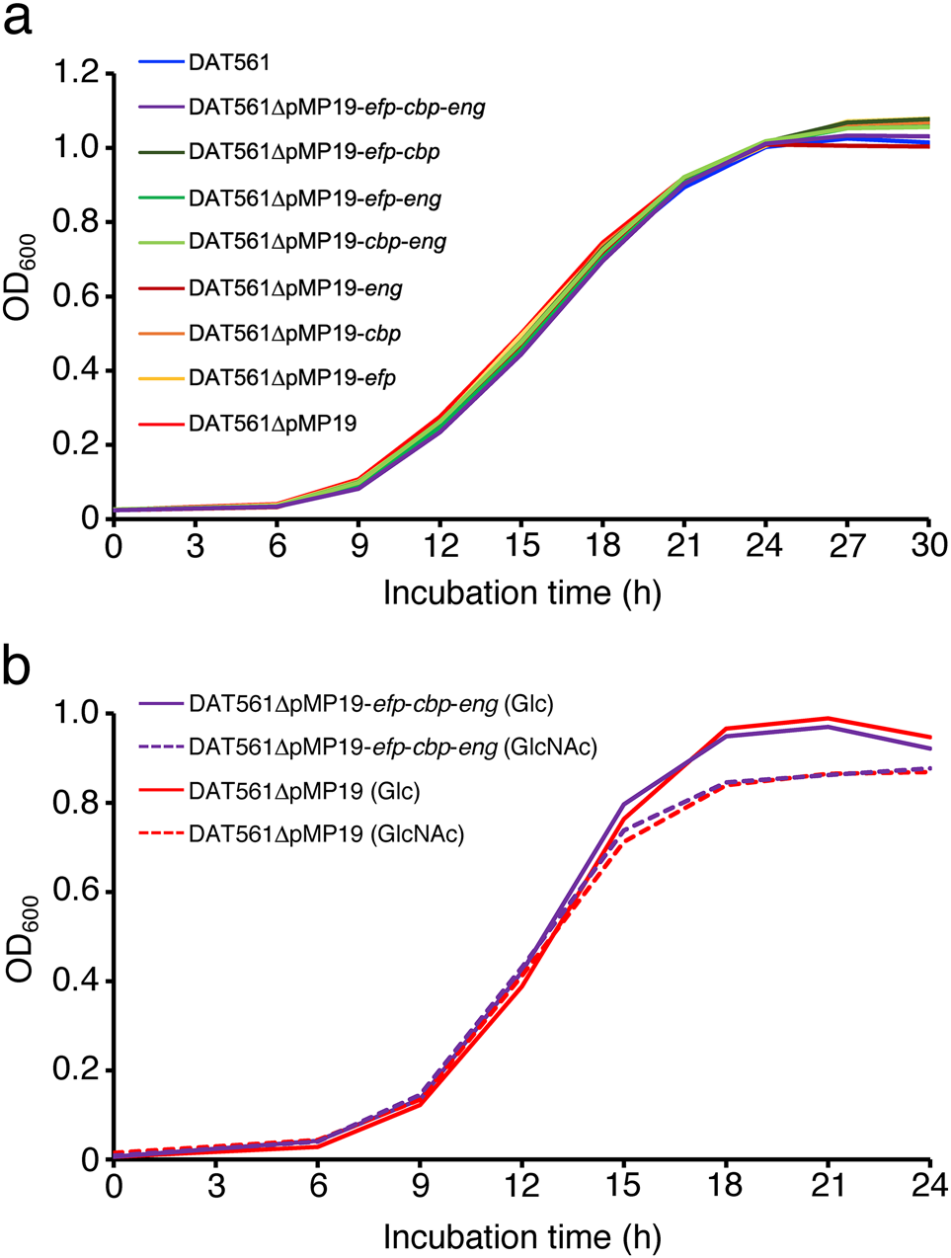
Growth curves of *M. plutonius* DAT561 and its derivatives in KSBHI broth (**a**) and carbohydrate test media supplemented with glucose (Glc) and *N*-acetylglucosamine (GlcNAc) (**b**). Growth of each strain in KSBHI broth was measured in duplicate in each test and the tests were repeated twice, whereas growth in carbohydrate test media was measured using five independent culture tubes. Means of the optical density at 600 nm (OD_600_) are shown.

In order to clarify the role of the three putative PM-degrading proteins in the pathogenesis of EFB caused by the highly virulent strain DAT561, we constructed single-, double- and triple-knockout mutants of the *efp*, *cbp* and *eng* genes from DAT561 (Table 2) as described in Methods using the primers listed in Supplementary Table S1. PCR and sequencing analyses confirmed that the *efp*, *cbp* and *eng* gene regions in the mutants were deleted (Fig. 2). The parent strain DAT561 possesses pMP19 (Fig. 2)^14^. Although the reason/mechanism is unknown, *M. plutonius* cells cannot maintain the plasmid stably during in vitro propagation^12,16^. As expected, all constructed mutants lost pMP19 during the repeated subcultures required for deleting the target genes (Fig. 2). Deletion of the putative PM-degrading protein genes had no significant effect on bacterial growth in vitro as shown in Fig. 3. Of note, although sequence analyses suggested the activity of the putative PM-degrading proteins to degrade crystalline chitin (Cbp) and mucin (Efp/endo-α-GalNAc-ase), *M. plutonius* was unable to utilize crystalline chitin and mucin as carbon sources even in the presence of the three genes (Supplementary Fig. S2), suggesting that the putative PM-degrading proteins cannot produce monosaccharides available to *M. plutonius* from the tested substrates and that additional enzymes are necessary to utilize the substrates as carbon sources. Indeed, in *E. faecalis*, both *Ef*Chi18A with endochitinase activity and *Ef*CBM33A, the homologue of *M. plutonius* Cbp, are necessary to depolymerize the insoluble chitinous substrate to soluble chitobiose. Then, the glycoside hydrolase 20 *N*-acetylhexosaminidase (or chitobiase) cleaves chitobiose to *N*-acetylglucosamine (GlcNAc), and GlcNAc is taken up by the bacterium via the GlcNAc-specific phosphotransferase sugar transporter^31^. However, in the genomes of *M. plutonius*, no *Ef*Chi18A homologue was found by the blastp program (https://blast.ncbi.nlm.nih.gov/Blast.cgi).

**Table 2:** *M. plutonius* strains used in this study.

Strain	Presence ( +) or absence (-)				References
	pMP19	Gene encoding			
		Enhancin	Chitin-binding domain-containing protein	Endo-α-*N*-acetylgalactosaminidase	
DAT561 (the parent strain)	+	+	+	+	^33^
DAT561ΔpMP19^a^	−	+	+	+	^16^
DAT561ΔpMP19-*efp*^a^	−	−	+	+	This study
DAT561ΔpMP19-*cbp*^a^	−	+	−	+	This study
DAT561ΔpMP19-*eng*^a^	−	+	+	−	This study
DAT561ΔpMP19-*efp*-*cbp*^b^	−	−	−	+	This study
DAT561ΔpMP19-*efp*-*eng*^b^	−	−	+	−	This study
DAT561ΔpMP19-*cbp*-*eng*^c^	−	+	−	−	This study
DAT561ΔpMP19-*efp*-*cbp*-*eng*^d^	−	−	−	−	This study

^a^Strains derived from DAT561.

^b^Strains derived from DAT561ΔpMP19-*efp.*

^c^Strain derived from DAT561ΔpMP19-*cbp.*

^d^Strain derived from DAT561ΔpMP19-*efp*-*cbp.*

### The putative PM-degrading protein gene deletion mutants were still virulent in honey bee broods

The virulence of the PM-degrading protein gene deletion mutants was evaluated using the in vitro* A. mellifera* larvae infection model as described in Methods and Supplementary Tables S2–S4. As all of the constructed mutants lost pMP19 (Fig. 2d), we compared the virulence of the mutants with that of the strain DAT561ΔpMP19 (DAT561 that lost pMP19) (Table 2)^16^.

**Figure 4:**
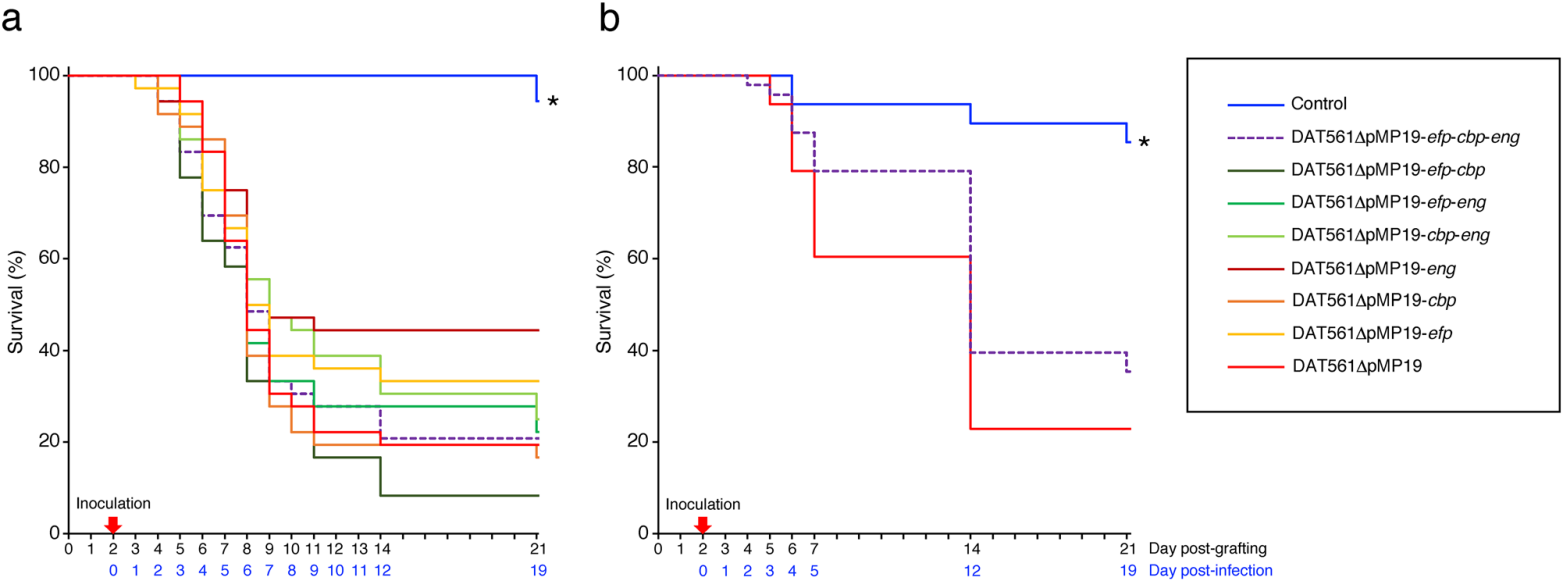
Evaluation of the virulence of *M. plutonius* DAT561ΔpMP19 and DAT561-derivative strains. The survival of *A. mellifera* larvae in Experimental Infections I (**a**) and II (**b**) is shown. *M. plutonius* strains and the number of larvae used in each group, and the infection doses are shown in Supplementary Table S4. All strains were cultured on KSBHI agar plates at 34.5 °C for 3–5 days under anaerobic conditions and used to prepare inocula containing 1.1–2.3 × 10^6^ CFU/ml of *M. plutonius*. The experiments were carried out as described in Methods. In Experimental Infection I (**a**), larvae (N = 36–72/group) were monitored daily until day 14 post-grafting (pg) and then at day 21 pg. In Experimental Infection II (**b**), larvae (N = 48/group) were monitored daily until day 7 pg, and then at days 14 and 21 pg. Differences in the survival rate of larvae were statistically analyzed by the log-rank test and Bonferroni *post-hoc* test using EZR^47^, which is a graphical user interface for the R software^48^. For comparison, *P* < 0.05 was considered significant. Asterisks indicate groups for which the survival was significantly different from that of the DAT561ΔpMP19-infected group.

As shown in Fig. 4a, more than 90% of the bees survived and became adults by day 21 post-grafting (pg) under the non-infected control conditions. When larvae were infected with DAT561ΔpMP19, the strain killed the larvae rapidly and the survival rate of the group at day 21 pg was only 19.4%. These results were consistent with our previous study^16^. As mentioned above, we expected the three putative PM-degrading proteins to be key virulence factors in the highly virulent *M. plutonius* strain. However, all constructed mutants were still virulent in honey bee broods. Even in the triple-knockout mutant (DAT561ΔpMP19-*efp*-*cbp*-*eng*)-infected group, the survival rate at day 21 pg was only 20.8%, almost the same level as in the DAT561ΔpMP19-infected group (Fig. 4a). Although the survival rate of the DAT561ΔpMP19-*eng*-infected group at day 21 pg was relatively high (44.4%), there was no significant difference in survival between each mutant and DAT561ΔpMP19 (log-rank test and Bonferroni *post-hoc* test, *P* = 1.0). As reported in the previous study by Lewkowski and Erler^10^, different results may be obtained when queens with different genetic backgrounds are used for in vitro assays. However, in this experiment, larvae from two different queens were used (Supplementary Table S4). Although the final concentration of *M. plutonius* in inocula slightly differed from strain to strain (1.1–1.5 × 10^6^ CFU/ml), the inoculation dose of DAT561ΔpMP19-*efp*-*cbp*-*eng* (1.1 × 10^6^ CFU/ml [i.e., 2.2 × 10^4^ CFU/larva]) was the same as that of DAT561ΔpMP19 (Supplementary Table S4). Therefore, our results strongly suggest that neither Efp, Cbp nor endo-α-GalNAc-ase is the indispensable virulence factor, at least in the CC12 strain DAT561. Of note, as all DAT561 mutants constructed and tested in this study lost pMP19, these results suggested again that major virulence factors of DAT561 are also not located on pMP19, although the same may not necessarily apply to all other virulent strains of CC12.

### The putative PM-degrading proteins are involved in PM degradation, but total degradation of the PM is dispensable in the pathogenesis of EFB caused by the highly virulent *M. plutonius* strain

In order to investigate whether the triple-knockout mutant can degrade the PM even in the absence of the three putative PM-degrading proteins, i.e., whether degradation and penetration of the PM are necessary for the highly virulent *M. plutonius* strain to kill larvae, we performed Experimental Infection II using DAT561ΔpMP19 and DAT561ΔpMP19-*efp*-*cbp*-*eng* (Supplementary Table S4), and collected control and infected larvae at days 2 and 4 post-infection [pi] (days 4 and 6 pg) for histopathological analyses.

**Figure 5:**
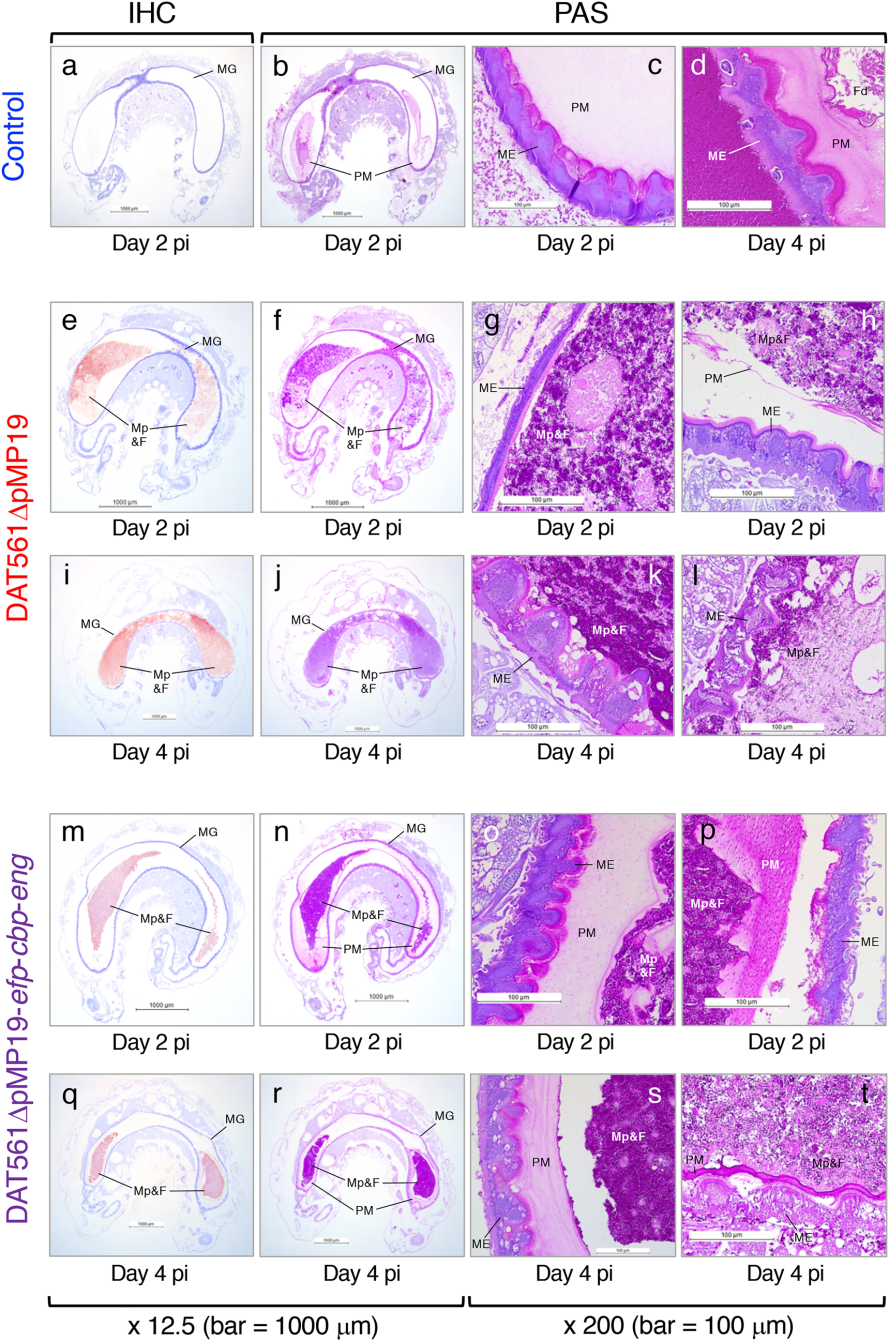
Light microscopic observations of representative non-infected control larvae [larva nos. 5 (**a**–**c**) and 8 (**d**)], DAT561ΔpMP19-infected larvae [larva nos. 18 (**e**–**g**), 15 (**h**), 22 (**i–k**) and 21 (**l**)] and DAT561ΔpMP19-*efp*-*cbp*-*eng*-infected larvae [larva nos. 31 (**m–o**), 36 (**p**), 44 (**q–s**) and 38 (**t**)] (refer to Supplementary Table S5 for information about each larva). Surviving larvae were collected at days 2 and 4 post-infection (pi) (days 4 and 6 post-grafting). The larvae were then fixed, dehydrated, embedded in paraffin and sectioned. The sections were stained using the periodic acid-Schiff (PAS) reaction and immunohistochemical (IHC) staining with rabbit polyclonal antiserum against *M. plutonius* cells^25^. MG, midgut; PM, peritrophic matrix; ME, midgut epithelial cells; Fd, brood foods in the midguts; Mp&F, *M. plutonius* and brood foods in the midguts.

In Experimental Infection II, 85.4% of the non-infected control broods became adult bees by day 21 pg (Fig. 4b). Consistent with the results of Experimental Infection I, DAT561ΔpMP19 killed larvae rapidly and the survival rate of the group at day 21 pg was 22.9%. Although the survival rate of bees infected with DAT561ΔpMP19-*efp*-*cbp*-*eng* at day 21 pg (35.4%) was slightly higher than that of the DAT561ΔpMP19-infected group, there was no significant difference in survival between the two groups (log-rank test and Bonferroni *post-hoc* test, *P* = 0.2). For histopathological analysis, we sampled only larvae that survived to the day of sampling (Supplementary Table S4), and the PM, midgut epithelial cells and *M. plutonius* in larvae were observed under a light microscope. The results are summarized in Supplementary Table S5, and representative micrographs of control and infected larvae are shown in Fig. 5.

In non-infected control larvae, ingested foods were surrounded by the PM and no *M. plutonius* was detected (Fig. 5a,b and larva nos. 1–8 in Supplementary Table S5a). Degeneration of the epithelial cells (i.e., cells with many vacuoles/foam in the cytoplasm or with granular cytoplasm) was absent or only rarely observed in most of the larvae (Fig. 5c,d and larva nos. 1–8 in Supplementary Table S5a).

In contrast, all of the DAT561ΔpMP19-infected larvae had immunohistochemical (IHC) staining-positive *M. plutonius* cells in the midgut (Fig. 5e,i and larva nos. 9–27 in Supplementary Table S5b). At day 2 pi (day 4 pg), proliferated *M. plutonius* cells in the midgut were confined by the PM in 5 of the 10 larvae analyzed (larva nos. 10 and 14–17 in Supplementary Table S5b). However, in the other larvae, the bacterial cells breached the PM and free *M. plutonius* directly contacted the midgut epithelial cells (Fig. 5e–g and larva nos. 9, 11–13 and 18 in Supplementary Table S5b). In larva no. 15, the *M. plutonius* mass was narrowly confined in the peritrophic sac, but the PM was thin and considered to be degraded (Fig. 5h). At day 4 pi (day 6 pg), the PM was disrupted or markedly degraded in most of the DAT561ΔpMP19-infected larvae, and *M. plutonius* cells were no longer surrounded by the PM (Fig. 5i–l and larva nos. 19–24, 26 and 27 in Supplementary Table S5b). In the larvae, highly proliferated *M. plutonius* cells directly contacted the midgut epithelial cells (Fig. 5k,l), and degenerated or disintegrated epithelial cells with many vacuoles/foam in the cytoplasm, granular/basophilic cytoplasm and/or granular/swollen/shrunken nuclei were observed throughout the midgut epithelium (Fig. 5k,l and larva nos. 19–27 in Supplementary Table S5b). In addition, as observed in both the parent strain (DAT561)- and DAT561ΔpMP19-infected larvae in the previous studies^16,33^, the larval growth was stunted in all DAT561ΔpMP19-infected larvae (the average weight at day 4 pi was 41.13 mg) compared with non-infected control larvae (the average weight at day 4 pi was 147.55 mg).

Many IHC-positive *M. plutonius* cells were also detected in the midgut of all the DAT561ΔpMP19-*efp*-*cbp*-*eng*-infected larvae, suggesting rapid proliferation of ingested *M. plutonius* in larvae (Fig. 5m,q and larva nos. 28–46 in Supplementary Table S5c). In all the larvae, the PM was partially detached from the midgut epithelium; however, unlike the DAT561ΔpMP19-infected group, the PM was abundant even at day 4 pi (day 6 pg) in most of the DAT561ΔpMP19-*efp*-*cbp*-*eng*-infected larvae. *M. plutonius* masses in the midguts were well confined by the PM and did not directly contact the midgut epithelial cells (Fig. 5n–p,r–t and larva nos. 28–46 in Supplementary Table S5c). As the PM became thin and/or had a fibrous or granular appearance in some larvae (e.g., Fig. 5p,t), the presence of PM-degrading factors other than Efp, Cbp and endo-α-GalNAc-ase cannot be excluded. However, our present study strongly suggests that all or some of the three putative PM-degrading proteins are necessary, at least for the tested *M. plutonius* strain DAT561 of CC12, to breach the peritrophic sac by day 4 pi. In the previous experiments by Aupperle-Lellbach et al.^26^, infection of the *M. plutonius* type strain did not affect the PM of honey bee larvae. According to the genome sequencing data, this strain possesses genes encoding intact Efp and Cbp, whereas endo-α-GalNAc-ase of this type strain is considered to be non-functional due to truncation of the C-terminal region, including the GalBD_like domain (Table 1). These and our present data suggest that endo-α-GalNAc-ase is the most important factor for *M. plutonius* to degrade the PM and breach the peritrophic sac in larvae, although further pathological analyses of larvae infected with the other single and double knockout mutants are needed to confirm this hypothesis.

Although the ability of DAT561ΔpMP19-*efp*-*cbp*-*eng* to breach the PM was impaired, the strain killed honey bee brood at a similar degree to DAT561ΔpMP19 (Fig. 4). In addition, in most of the triple-knockout mutant-infected larvae, many degenerated or disintegrated epithelial cells were detected at day 4 pi (day 6 pg) (Fig. 5s,t and larva nos. 38–44 in Supplementary Table S5c). Furthermore, as with DAT561ΔpMP19-infected larvae, the growth of larvae infected with the mutant was stunted at day 4 pi (the average weight was 62.01 mg). This suggests that total degradation of the PM is dispensable for the highly virulent strain to kill larvae, supporting that neither Efp, Cbp nor endo-α-GalNAc-ase is the indispensable virulence factor, at least in the *M. plutonius* CC12 strain.

In AFB, degradation of the PM by *Pl*CBP49 is considered to enable access of *P. larvae* to gut epithelial cells and to help meet nutritional needs, i.e., the degradation enables *P. larvae* to use chitin as an additional carbon source in larvae^20,21^. Therefore, although deletion of the putative PM-degrading protein genes had no significant effect on *M. plutonius* growth in artificial culture media (Fig. 3), growth of the triple knockout *M. plutonius* in larvae may have been affected due to impaired ability to degrade the PM and shortage of carbon source supply from the PM. However, as shown in Supplementary Fig. S2, DAT561ΔpMP19 was unable to utilize crystalline chitin and mucin as carbon sources at least in in vitro culture tubes. In addition, although not quantitatively measured, a large quantity of IHC-positive *M. plutonius* cells was observed in the triple knockout mutant-infected larvae (Fig. 5m,q and Supplementary Table S5c). Therefore, unlike *P. larvae*, *M. plutonius* may not use the PM as carbon sources in larvae. As there was no significant difference in survival between DAT561ΔpMP19 and DAT561ΔpMP19-*efp*-*cbp*-*eng*-infected groups (Fig. 4), even if growth of DAT561ΔpMP19-*efp*-*cbp*-*eng* was slightly impaired in larvae, its impact on the pathogenicity of *M. plutonius* CC12 strains is considered negligible under the conditions of the experimental infections. However, under the experimental conditions, as larvae were fed artificial diets containing lots of monosaccharides easily available to *M. plutonius* (Supplementary Table S2), impacts of the three proteins on the virulence of *M. plutonius* may have been underestimated. Therefore, further studies including optimization of artificial diets and detailed comparison of growth ability between wild-type and PM-degrading protein gene-deficient *M. plutonius* strains in infected larvae will be needed for a deeper understanding of the role of the putative PM-degrading proteins in larvae.

So, why did the DAT561ΔpMP19-*efp*-*cbp*-*eng*-infected larvae die under the experimental conditions tested in this study? As mentioned above, degeneration of the PM was observed even in the DAT561ΔpMP19-*efp*-*cbp*-*eng*-infected larvae (e.g., Fig. 5p,t). The integrity of the PM is highly important for normal larval development and survival^20^. When honey bee larvae were fed a diet containing Calcofluor White, a stilbene derivative with chitin binding properties that interferes with chitin fibrillogenesis^34^, dissociates chitin-bound proteins from the PM in vitro and block PM formation in vivo^35^ and destroys the PM by inducing pores^36^, larval mortality significantly increased in the absence of a notable effect on PM integrity (2.5 mM Calcofluor White), demonstrating that disturbed PM integrity is sufficient to cause larval mortality even in the absence of a histologically visible effect on PM formation^20^. In the DAT561ΔpMP19-*efp*-*cbp*-*eng*-infected larvae, disturbance of PM integrity and/or PM formation by unidentified *M. plutonius* factors may have affected the normal larval development and survival.

In most of the DAT561ΔpMP19-*efp*-*cbp*-*eng*-infected larvae, degenerated or disintegrated epithelial cells were also observed at day 4 pi (e.g., Fig. 5s,t). In another insect pathogen, *Bacillus thuringiensis*, *Bacillus* enhancin-like protein produced by the bacterium degraded the insect intestinal mucin of the larval PM and increased insecticidal activity of the *B. thuringiensis* crystal protein toxin against *Helicoverpa armigera* larvae^37^. Therefore, in the triple knockout mutant-infected larvae, PM degeneration by unidentified factors may have increased PM permeability, thereby facilitating passage of bacterial toxins through the PM to attack the underlying epithelium. In *M. plutonius*, melissotoxin A encoded in the virulence plasmid pMP19 was found as a putative insecticidal toxin of this bacterium. However, as the triple knockout mutant lost pMP19 during the process of gene knockout, other factors (presumably relatively small molecules that can permeate the PM easily) may have affected the integrity of the midgut epithelial cells. Previous genome analysis by Djukic et al.^12^ identified an *Enterococcus*-type tyrosine decarboxylase gene cluster, which is involved in tyramine production. Kanbar et al.^38,39^ reported that tyramine production of *E. faecalis* has highly toxic effects on honey bee larvae leading to classical EFB-like symptoms. Therefore, tyramine produced by DAT561ΔpMP19-*efp*-*cbp*-*eng* may be one of the factors that permeates the PM and damages the midgut epithelial cells. Of note, the genes encoding tyrosine decarboxylase are putatively non-functional due to a nonsense mutation in the type strain of *M. plutonius* (LMG 20360, ATCC 35311)^12^, which did not destroy the intestinal epithelium in infected larvae^26^. These reports suggest the importance of tyramine production in EFB.

This study demonstrated that all or some of the three putative PM-degrading proteins of *M. plutonius* are involved in degradation of the PM in honey bee larvae. As larval mortality is known to increase by only disturbing the PM integrity^20^, the putative PM-degrading proteins, which can actually degrade the PM, probably function as virulence factors of *M. plutonius*. However, as deletion of the PM-degrading protein genes did not decrease larval mortality by the highly virulent CC12 strain, neither of the three proteins was considered as the indispensable virulence factor in the CC12 strain at least in the in vitro* A. mellifera* larvae infection model. However, these proteins may interact with other genes/proteins or are part of a cascade that promotes pathogenesis and the distribution of EFB under colony conditions. In this study, although we focused on only the pathomechanisms of EFB caused by the virulent CC12 strain, the reason why some CC13 strains including DAT585 and LMG 20360 are low virulent is also interesting. As strain LMG 20360 lost the ability to degrade the PM^26^, the virulence of CC13 strains may increase by repairing mutations in the PM-degrading protein genes. However, to verify these hypotheses and elucidate the pathogenesis of EFB caused by different *M. plutonius* strains, further studies of the role of the putative PM-degrading proteins at the colony level and identification of other virulence factors will be necessary.

## Conclusions

The rapid advances in genome sequencing technologies are providing vast quantities of nucleotide sequence data and many putative virulence factors have been found in many pathogens. However, the true roles of the factors in the pathogenesis of infectious diseases cannot be determined by only in silico analyses, and it is essential to analyze live bacteria, including isogenic mutants, in addition to evaluating in silico data. Indeed, although the genes encoding Efp, Cbp and endo-α-GalNAc-ase were described as putative virulence factors in the previous comparative genomic study of *M. plutonius* strains^12^, their roles in the pathogenesis of EFB were unknown due to a lack of data from isogenic mutants of the genes. In this study, we constructed a series of putative PM-degrading protein gene knockout mutants from the highly virulent CC12 strain DAT561, and demonstrated that *M. plutonius* DAT561 can kill honey bee larvae even in the absence of the three genes. As the plasmid pMP19 plays a significant role in the virulence of *M. plutonius* strains only in CC3^16^, we cannot exclude the possibility that the three putative PM-degrading proteins are also essential for *M. plutonius* strains with specific genotypes to kill honey bee larvae aggressively; however, our present study suggested that the three proteins were dispensable virulence factors at least in *M. plutonius* DAT561, and demonstrated the usefulness and importance of our genetic tools and techniques^16,40–42^ for evaluating the roles of *M. plutonius* genes in the pathogenesis of EFB. In this study, we were unable to find indispensable virulence factors in the highly virulent *M. plutonius* strain; however, the existence of virulence factors that can go through the PM and affect the integrity of the PM and/or midgut epithelial cells in honey bee larvae was suggested. The previous comparative genomic analysis^12^ detected many other virulence factor candidates, including bacteriocins, bacteria cell surface- and host cell adhesion-associated proteins, an enterococcal polysaccharide antigen, an enzyme involved in tyramine production and capsule-associated proteins. We are currently performing experiments to elucidate the role of these putative virulence factors using genetic tools and techniques for *M. plutonius*, in addition to the in vitro* A. mellifera* larvae infection model.

## Methods

### Bacterial strains, culture conditions and plasmid vector

*Melissococcus plutonius* strains used in this study are listed in Table 2, and the thermosensitive plasmid vector pSET6s, which has multiple cloning sites in the *lacZ´* gene^40^, was used to construct gene knockout vectors for gene manipulation of *M. plutonius* strains. *E. coli* TOP10 (Invitrogen, Carlsbad, CA, USA) was used to host the constructed vectors. *M. plutonius* and *E. coli* strains were cultured using KSBHI broth or agar^9,33^ at 34.5–35 °C under anaerobic conditions and LB (Becton, Dickinson and Company, Franklin Lakes, NJ, USA) broth or agar at 37 °C under aerobic conditions, respectively, as described previously^16^. For maintenance of the plasmid vectors, chloramphenicol was added to the media at the following concentrations: 8–15 µg/ml for *E. coli* and 4–15 µg/ml for *M. plutonius*. For selection of *E. coli* strains with pSET6s-based recombinant plasmids by the blue-white screening technique, 5-bromo-4-chloro-3-indolyl-β-D-galactopyranoside was added to LB plates at 100 µg/ml. Bacterial DNA was prepared using InstaGene Matrix (Bio-Rad Laboratories, Inc., Hercules, CA, USA) in accordance with the manufacturer’s instructions.

### Amino acid sequence analysis of the putative PM-degrading proteins

Amino acid sequences of the putative PM-degrading proteins of *M. plutonius* strains with different genetic backgrounds, *Pl*CBP49 of *P. larvae* ATCC 9545, CBP21 of *S. marcescens* and *Ef*CBM33A of *E. faecalis* were retrieved from the GenBank database at the National Center for Biotechnology Information (https://www.ncbi.nlm.nih.gov/). Multiple sequence alignments of the proteins were computed using ClustalW (https://clustalw.ddbj.nig.ac.jp/). Signal peptides were predicted by SignalP-5.0 (http://www.cbs.dtu.dk/services/SignalP/).

### Construction of *M. plutonius* gene deletion mutants

For construction of precise in-frame deletions in the *efp*, *cbp* and *eng* genes, upstream and downstream regions of the genes were amplified from genomic DNA of the *M. plutonius* strain DAT561 by PCR, and fused by overlap-extension PCR^41^ using the primers and enzymes listed in Supplementary Table S1. The resulting PCR products were cloned into the EcoRI or PstI site of the thermosensitive plasmid vector pSET6s^40^ in *E. coli* TOP10 (Invitrogen). The resulting plasmids were introduced into *M. plutonius* DAT561 or DAT561 derivatives (DAT561ΔpMP19-*efp*, DAT561ΔpMP19-*cbp* and DAT561ΔpMP19-*efp*-*cbp*) by electroporation^42^, and double-crossover gene deletion mutants were generated according to the procedures described previously^41^. Deletion of the target genes was confirmed by PCR using the primers and enzymes listed in Supplementary Table S1 and sequencing analysis of the amplified products.

### *Melissococcus plutonius* growth measurements

KSBHI broth^9^ and carbohydrate test media supplemented with four different carbon sources (glucose [FUJIFILM Wako Pure Chemical Corp., Osaka, Japan], GlcNAc [FUJIFILM Wako], chitin [FUJIFILM Wako] and mucin [Difco Laboratories, Becton, Dickinson and Company]) (Supplementary Table S6) were used to compare the growth of DAT561 and the derivatives. Strains were streaked onto KSBHI agar plates and incubated at 35 °C for five days under anaerobic conditions. Bacteria were then suspended in KSBHI broth and suspension medium (Supplementary Table S6) to an optical density at 600 nm (OD_600_) of 0.4 and 0.6 using an SP-300 spectrophotometer (OPTIMA, Tokyo, Japan) for growth measurements in KSBHI broth and carbohydrate test media, respectively. After adding 1/40 of the volume of each adjusted bacterial suspension to the media, the cultures were incubated at 35 °C under anaerobic conditions. The OD_600_ of the cultures was measured at 0, 6, 9, 12, 15, 18, 21, 24, 27 and/or 30 h after incubation. Growth of each strain in KSBHI broth was measured in duplicate in each test and the tests were repeated twice, whereas growth in carbohydrate test media was measured using five independent culture tubes. To confirm acid production from supplemented carbon sources, bromocresol purple (FUJIFILM Wako) was added to the suspension and carbohydrate test media to a final concentration of 0.003% (w/v), and a color change of the media was observed after a 36-h incubation.

### Preparation of inocula for experimental infection

Inocula used for experimental infection were prepared as described previously^16^. Briefly, *M. plutonius* strains cultured on KSBHI agar plates^33^ were suspended in sterile H_2_O at a final concentration of approximately 1–2 × 10^7^ CFU/ml, and the suspension and artificial diet B' (Supplementary Table S2) were mixed at a ratio of 1:9. The final bacterial concentration in each inocula measured as described previously^16^ are shown in Supplementary Table S4.

### Experimental infection

Clinically healthy *A. mellifera* colonies were purchased from several different apiaries, and less than 24-h-old larvae were collected from 2–3 different queens for each experiment (Supplementary Table S4) and randomly divided into test groups. The larvae were infected with *M. plutonius* strains at day 2 pg by feeding 20 µl of artificial diet containing *M. plutonius* (inocula) and reared using 48-well cell culture plates until day 21 pg as described previously^16^. Larvae in non-infected control groups were fed normal artificial diet (diet B in Supplementary Table S2) at day 2 pg. Formulas and daily rations of the artificial diet are shown in Supplementary Tables S2 and S3, respectively, and we confirmed that the royal jelly used to prepare the artificial diets did not contain live *M. plutonius* as described previously^16^. In Experimental Infection I, larvae were monitored daily until day 14 pg and then at day 21 pg. In Experimental Infection II, two groups of larvae were tested for each *M. plutonius* strain. One group was used to assess survival rate and another was used to collect larval samples for histopathological analysis. In the survival rate group of Experimental Infection II, larvae were monitored daily until day 7 pg, and then at days 14 and 21 pg. The total number of larvae used for each experiment is shown in Supplementary Table S4.

### Histopathological analysis

Surviving larvae were collected at days 2 and 4 pi (days 4 and 6 pg) for histopathological analysis. The larvae were then rinsed for several seconds with sterilized distilled water, fixed in 10% phosphate-buffered formalin, dehydrated in a series of ethanol, embedded in paraffin and sectioned using standard histological techniques^43–46^. The sections were stained with PAS reaction using Cold Schiff’s Reagent (FUJIFILM Wako) according to the manufacturer’s instructions. IHC staining was also performed using commercial kits (Histofine Simple Stain MAX-PO (MULTI) and Histofine Simple Stain AEC Solution, Nichirei Biosciences Inc., Tokyo, Japan) according to the manufacturer’s instructions. Rabbit polyclonal antiserum against formalin-fixed *M. plutonius* cells^25^ was diluted 8,192-fold in IHC diluent (primary Ab) (Enzo Life Sciences, Inc., Farmingdale, NY, USA) and used as the primary antibody to detect the antigen. The stained larval sections were observed under a light microscope (Leica DM2000; Leica, Wetzlar, Germany).

### Statistical analysis

The differences in the survival of tested larvae throughout the experiments were analyzed by the log-rank test and Bonferroni *post-hoc* test, and a value of *P* < 0.05 was set as the threshold for significance. All statistical analyses were performed using EZR (Saitama Medical Center, Jichi Medical University, Saitama, Japan)^47^, which is a graphical user interface for the R software^48^.

## Supplementary Information

Supplementary Information.

## Data Availability

The datasets generated during the current study are available from the corresponding author upon reasonable request.
