# Supplementary material for: Peritrophic matrix-degrading proteins are dispensable virulence factors in a virulent Melissococcus plutonius strain
Source: Sci Rep. 2021 Apr 22;11:8798. doi: 10.1038/s41598-021-88302-8 (PMC8062581; doi:10.1038/s41598-021-88302-8)
Supplement: Supplementary file 1 — Supplementary Information. [file 41598_2021_88302_MOESM1_ESM.pdf]

Supplementary Information for

Peritrophic matrix-degrading proteins are dispensable virulence factors in a virulent *Melissococcus plutonius* strain

Keiko Nakamura[1], Kayo Okumura[2], Mariko Harada[1], Mariko Okamoto[3], Masatoshi Okura[3], Daisuke Takamatsu[3,4]

[1] Research and Business Promotion Division, Research Institute for Animal Science in Biochemistry and Toxicology, Sagamihara, Kanagawa 252-0132, Japan

[2] Department of Veterinary Medicine, Obihiro University of Agriculture and Veterinary Medicine, Obihiro, Hokkaido 080-8555, Japan

[3] Division of Bacterial and Parasitic Disease, National Institute of Animal Health, National Agriculture and Food Research Organization, Tsukuba, Ibaraki 305-0856, Japan

[4] The United Graduate School of Veterinary Sciences, Gifu University, Gifu, Gifu 501-1193, Japan

Supplementary Table S1. PCR primers used in the present study.

Primer	Sequence (5'-3')	Description	Polymerase used for PCR	PCR program
**For construction of enhancin family protein gene (*efp*) knockout vector**				
efp-KO1	AGCACTCTAGCCATGAATCA	For amplification of upstream region of *efp* in 1st PCR.		
efp-KO2	TTTCCCCTTGCTCATTCCAG	For amplification of upstream region of *efp* in 1st PCR.		
efp-KO3	ATTGTTGAGGGTGACATGGT	For amplification of downstream region of *efp* in 1st PCR.		1st PCR (efp-KO1+efp-KO2/efp-KO3+efp-KO4)
efp-KO4	CCGTCACTATTTGGTAAACT	For amplification of downstream region of *efp* in 1st PCR.		98°C 2 min - 98°C 10 sec, 57°C 20 sec, 72°C 1 min (30 cycles) - 72°C 2 min
efp-KO5	GAACGAATTCCAATCTATAATCGAGGACAC	For amplification of upstream region of *efp* in 2nd PCR and generation of the fused fragment in 3rd PCR. EcoRI site is underlined.	iProof (Bio-Rad)	2nd PCR (efp-KO5+efp-KO6/efp-KO7+efp-KO8)<br>98°C 2 min - 98°C 10 sec, 55°C 20 sec, 72°C 1 min (30 cycles) - 72°C 2 min
efp-KO6	CTCTCTCTTTTGCACCTGATAAGCACCTCC	For amplification of upstream region of *efp* in 2nd PCR.		3rd PCR (efp-KO5+efp-KO8)
efp-KO7	GGAGGTGCTTATCAGGTGCAAAAGAGAGAG	For amplification of downstream region of *efp* in 2nd PCR.		98°C 2 min - 98°C 10 sec, 55°C 20 sec, 72°C 1 min 30 sec (30 cycles) - 72°C 3 min
efp-KO8	ATTAGAATTCATGCAAGCTTTATGGCATAG	For amplification of downstream region of *efp* in 2nd PCR and generation of the fused fragment in 3rd PCR. EcoRI site is underlined.		
**For confirmation of the *efp* gene deletion in the constructed mutants**				
efp-KO1	AGCACTCTAGCCATGAATCA	For amplification of the *efp* gene region.	KOD FX (TOYOBO)	94°C 2 min - 98°C 10 sec, 55°C 30 sec, 68°C 4 min (30 cycles) -68°C 5 min
efp-KO4	CCGTCACTATTTGGTAAACT			
**For construction of chitin-binding domain-containing protein gene (*cbp*) knockout vector**				
cbp-KO1	TGTAAGTCTAGATTGCCACC	For amplification of upstream region of *cbp* in 1st PCR.		
cbp-KO2	ACATAGCCATGTGCAGACAC	For amplification of upstream region of *cbp* in 1st PCR.		
cbp-KO3	TTCAGTGTGGGATATTAACG	For amplification of downstream region of *cbp* in 1st PCR.		1st PCR (cbp-KO1+cbp-KO2/cbp-KO3+cbp-KO4)
cbp-KO4	TTGGTAAAGGCTGCTAATGG	For amplification of downstream region of *cbp* in 1st PCR.		98°C 1 min - 98°C 10 sec, 55°C 20 sec, 72°C 1 min (30 cycles) - 72°C 3 min
cbp-KO5	GTTACTGCAGATGGTTTAAAGACAGAATTC	For amplification of upstream region of *cbp* in 2nd PCR and generation of the fused fragment in 3rd PCR. PstI site is underlined.	iProof (Bio-Rad)	2nd PCR (cbp-KO5+cbp-KO6/cbp-KO7+cbp-KO8)<br>98°C 1 min - 98°C 10 sec, 55°C 20 sec, 72°C 30 sec (30 cycles) - 72°C 3 min
cbp-KO6	TACATCAATGGCTTGTTTCATTTTGCCCTC	For amplification of upstream region of *cbp* in 2nd PCR.		3rd PCR (cbp-KO5+cbp-KO8)
cbp-KO7	GAGGGCAAAATGAAACAAGCCATTGATGTA	For amplification of downstream region of *cbp* in 2nd PCR.		98°C 1 min - 98°C 10 sec, 58°C 20 sec, 72°C 1 min (30 cycles) - 72°C 3 min
cbp-KO8	TATTCTGCAGCCATTCAAATCACCGATACC	For amplification of downstream region of *cbp* in 2nd PCR and generation of the fused fragment in 3rd PCR. PstI site is underlined.		
**For confirmation of the *cbp* gene deletion in the constructed mutants**				
cbp-KO1	TGTAAGTCTAGATTGCCACC	For amplification of the *cbp* gene region.	KOD FX (TOYOBO)	94°C 2 min - 98°C 10 sec, 68°C 4 min (30 cycles) -68°C 5 min
cbp-KO4	TTGGTAAAGGCTGCTAATGG			
**For construction of endo-α-*N*-acetylgalactosaminidase gene (*eng*) knockout vector**				
eng-KO1	GTTGCTACTGATTATGATGG	For amplification of upstream region of *eng* in 1st and 2nd PCR.		
eng-KO2	ATGCGCATACAATGAACAAG	For amplification of upstream region of *eng* in 1st PCR.		1st PCR (eng-KO1+eng-KO2/eng-KO3+eng-KO4)<br>98°C 2 min - 98°C 10 sec, 55°C 20 sec, 72°C 1 min (30 cycles) - 72°C 2 min
eng-KO3	ATACAGCAGGTGGTGCATAC	For amplification of downstream region of *eng* in 1st PCR.		
eng-KO4	CTTTCTTCTCTATCCGTTTC	For amplification of downstream region of *eng* in 1st and 2nd PCR.		2nd PCR (eng-KO1+eng-KO6/eng-KO4+eng-KO7)
eng-KO5	AAGGCTGCAGTGAATGAAGTAGGATTA	For generation of the fused fragment in 3rd PCR. PstI site is underlined.	iProof (Bio-Rad)	98°C 2 min - 98°C 10 sec, 55°C 20 sec, 72°C 1 min (30 cycles) - 72°C 2 min
eng-KO6	TCAATCTTTTGAGACCATTTTTAATCCTCC	For amplification of upstream region of *eng* in 2nd PCR.		3rd PCR (eng-KO5+eng-KO8)
eng-KO7	GGAGGATTAAAAATGGTCTCAAAAGATTGA	For amplification of downstream region of *eng* in 2nd PCR.		98°C 2 min - 98°C 10 sec, 55°C 20 sec, 72°C 1 min 30 sec (30 cycles) - 72°C 3 min
eng-KO8	ATACCTGCAGACCAATCAAATAAACCCATC	For generation of the fused fragment in 3rd PCR. PstI site is underlined.		
**For confirmation of the *eng* gene deletion in the constructed mutants**				
eng-KO1	GTTGCTACTGATTATGATGG	For amplification of the *eng* gene region.	KOD FX (TOYOBO)	94°C 2 min - 98°C 10 sec, 55°C 30 sec, 68°C 4 min (30 cycles) -68°C 5 min
eng-KO4	CTTTCTTCTCTATCCGTTTC			
**For detection of pMP19**				
pMP19S25	ACAACATCTACAAGTAATACC	For detection of pMP19 from *M. plutonius* strains.	Ex Taq DNA polymerase (Takara)	95°C 2 min - 95°C 20 sec, 55°C 20 sec, 72°C 30 sec (35 cycles) -72°C 2 min
pMP19R1	GACCATGTTACCCACAATCC			

Supplementary Table S2. Formulas of the artificial diet for honey bee larvae used in the experimental infections.

Contents	Artificial diet			
	A	B	B'	C
D-glucose	6 g	7.5 g	7.5 g	9 g
D-fructose	6 g	7.5 g	7.5 g	9 g
Yeast extract	1 g	1.5 g	1.5 g	2 g
Royal jelly	50 g	50 g	50 g	50 g
Sterile H_2O	37 g	33.5 g	23.5 g	30 g
Total	100 g	100 g	90 g	100 g

Supplementary Table S3. Daily rations of the artificial diet[a].

Day post-grafting	Artificial diet	Amount (μl)/larva
0	Diet A	20
2	Diet B (control group)/Inocula (infected group)	20
3	Diet C	30
4	Diet C	40
5	Diet C	50

[a] Larvae were not fed on day 6 post-grafting. Formulas of artificial diet are listed in Supplementary Table S2, and the inocula were made according to the recipe described in Methods.

Supplementary Table S4. Conditions of experimental infections performed in this study.

Experimental Infection	*M. plutonius* strain	pMP19[a]	Gene encoding[a]			Measurement items	No. of larvae used (from N queens)	Final concentration of *M. plutonius* in inocula (CFU/ml)
			Enhancin	Chitin-binding domain-containing protein	Endo-α-*N*-acetylgalactosaminidase			
I	Non-infected control	NA	NA	NA	NA	Survival	36 (2)	0
	DAT561ΔpMP19	-	+	+	+	Survival	36 (2)	1.1×10^6
	DAT561ΔpMP19-*efp*	-	-	+	+	Survival	36 (2)	1.2×10^6
	DAT561ΔpMP19-*cbp*	-	+	-	+	Survival	36 (2)	1.4×10^6
	DAT561ΔpMP19-*eng*	-	+	+	-	Survival	36 (2)	1.3×10^6
	DAT561ΔpMP19-*efp* -*cbp*	-	-	-	+	Survival	36 (2)	1.5×10^6
	DAT561ΔpMP19-*efp* -*eng*	-	-	+	-	Survival	36 (2)	1.2×10^6
	DAT561ΔpMP19-*cbp* -*eng*	-	+	-	-	Survival	36 (2)	1.1×10^6
	DAT561ΔpMP19-*efp* -*cbp* -*eng*	-	-	-	-	Survival	72 (2)	1.1×10^6
II	Non-infected control	NA	NA	NA	NA	Survival	48 (3)	0
						Histopathological analysis	8[b] (3)	0
	DAT561ΔpMP19	-	+	+	+	Survival	48 (3)	2.3×10^6
						Histopathological analysis	19[b] (3)	2.3×10^6
	DAT561ΔpMP19-*efp* -*cbp* -*eng*	-	-	-	-	Survival	48 (3)	2.1×10^6
						Histopathological analysis	19[b] (3)	2.1×10^6

[a] +, presence; -, absence; NA, not applicable.

[b] The number of larvae analyzed histopathologically. We reared more larvae than the numbers listed in this table for histopathological analysis. Among them, we sampled only surviving larvae for the analysis.

Supplementary Table S5. Summarized microscopic observations of control and infected larvae stained by the periodic acid-Schiff reaction and anti-*M. plutonius* rabbit serum.

Table S5a. Control larvae.

Days post-infection (pi)	Day 2 pi					Day 4 pi		
Larva no.	1	2	3	4	5	6	7	8
M. plutonius in the midgut[a]	-	-	-	-	-	-	-	-
Peritrophic matrix[a]	++	++	+	++	++	+	+	+
Degenerated midgut epithelial cells[b]	-	±	-	+	±	-	-	-

Table S5b. DAT561ΔpMP19-infected larvae.

Days post-infection (pi)	Day 2 pi										Day 4 pi								
Larva no.	9	10	11	12	13	14	15	16	17	18	19	20	21	22	23	24	25	26	27
M. plutonius in the midgut[a]	++	+	++	+++	++	++	+++	+	++	+++	+++	++	+++	+++	++	+++	+	+++	+++
Peritrophic matrix (PM)[a]	+/±[d]	+	+	+	±	+++	+	+	+++	+	±	±	+	±	+	±	+++	++	+
Confinement of *M. plutonius* by the PM[c]	**NC**	**C**	**NC**[e]	**NC**	**NC**	**C**	**C**[g]	**C**	**C**	**NC**[e]	**NC**	**NC**	**NC**	**NC**	**NC**	**NC**	**C**	**NC**	**NC**
Degenerated midgut epithelial cells[b]	±	+	+	++	+++[f]	±	++	-	-	±	+++	+++	+++	+++	++	+++[f]	++	++	++

Table S5c. DAT561ΔpMP19-*efp*-*cbp*-*eng*-infected larvae.

Days post-infection (pi)	Day 2 pi										Day 4 pi								
Larva no.	28	29	30	31	32	33	34	35	36	37	38	39	40	41	42	43	44	45	46
M. plutonius in the midgut[a]	++	++	++	+++	++	++	++	+	++	+	+++	++	++	+++	++	++	+++	++	+
Peritrophic matrix (PM)[a]	+++	+++	+++	+++	+++	+	+++	++	+++	++	+	+++	++	+++	++	++	++	+++	++
Confinement of *M. plutonius* by the PM[c]	**C**	**C**	**C**	**C**	**C**	**C**	**C**	**C**	**C**	**C**	**C**[g]	**C**	**C**	**C**	**C**	**C**	**C**	**C**	**C**
Degenerated midgut epithelial cells[b]	-	-	±	±	-	-	+	±	+	++	+++	++	++	++	+++	++	+++	±	±

[a] -, none; ±, rarely present; +, present; ++, abundant; +++, markedly abundant.

[b] Degenerated midgut epithelial cells were cells with many vacuoles/foam in the cytoplasm, granular/basophilic cytoplasm and/or granular/swollen/shrunken nuclei.
-, the midgut epithelium was almost intact.
±, degenerated epithelial cells were rarely present.
+, degenerated epithelial cells were observed in some places.
++, degenerated epithelial cells were abundantly present.
+++, almost all epithelial cells were degenerated.

[c] A mass of *M. plutonius* cells in the midgut was **confined** (**C**) or **not confined** (**NC**) by the PM.

[d] The midgut cut in round slices was observed in two places in the larval body. In one place where only a small number of *M. plutonius* cells were present, the PM was observed. In the other area of the midgut, abundantly observed *M. plutonius* cells were not surrounded by the PM, and some cells directly contacted the midgut epithelial cells.

[e] Although most of the *M. plutonius* cells were confined by the thin PM, some cells directly contacted the midgut epithelium.

[f] In a part of the midgut, the epithelium disappeared/was broken, and a mass of *M. plutonius* cells protruded from the midgut into the hemocoel.

[g] A mass of *M. plutonius* cells in the midgut was narrowly confined by the thin PM.

Table S6. Formulas of suspension and carbohydrate test media used in this study[a].

Contents	Suspension medium	Carbohydrate test media supplemented with			
		Glucose	GlcNAc[b]	Chitin[c]	Mucin
Yeast Extract (Bacto)	10	10	10	10	10
L-cysteine (FUJIFILM Wako)	0.25	0.25	0.25	0.25	0.25
KH_2PO_4 (Nacalai tesque)	13.6	13.6	13.6	13.6	13.6
Glucose (FUJIFILM Wako)	-	10	-	-	-
GlcNAc[b] (FUJIFILM Wako)	-	-	10	-	-
Chitin (FUJIFILM Wako)	-	-	-	10	-
Mucin (Difco)	-	-	-	-	10

[a] Unit: g/L. The pH was adjusted to 6.6 with KOH, and the media were autoclaved at 115°C for 10 min. To confirm acid production from supplemented carbon sources, bromocresol purple (FUJIFILM Wako) was added to the media to a final concentration of 0.003% (w/v).

[b] *N*-Acetyl-D(+)-glucosamine.

[c] Reduced L-glutathione (Sigma-Aldrich) was added to the medium to a final concentration of 1 mM.

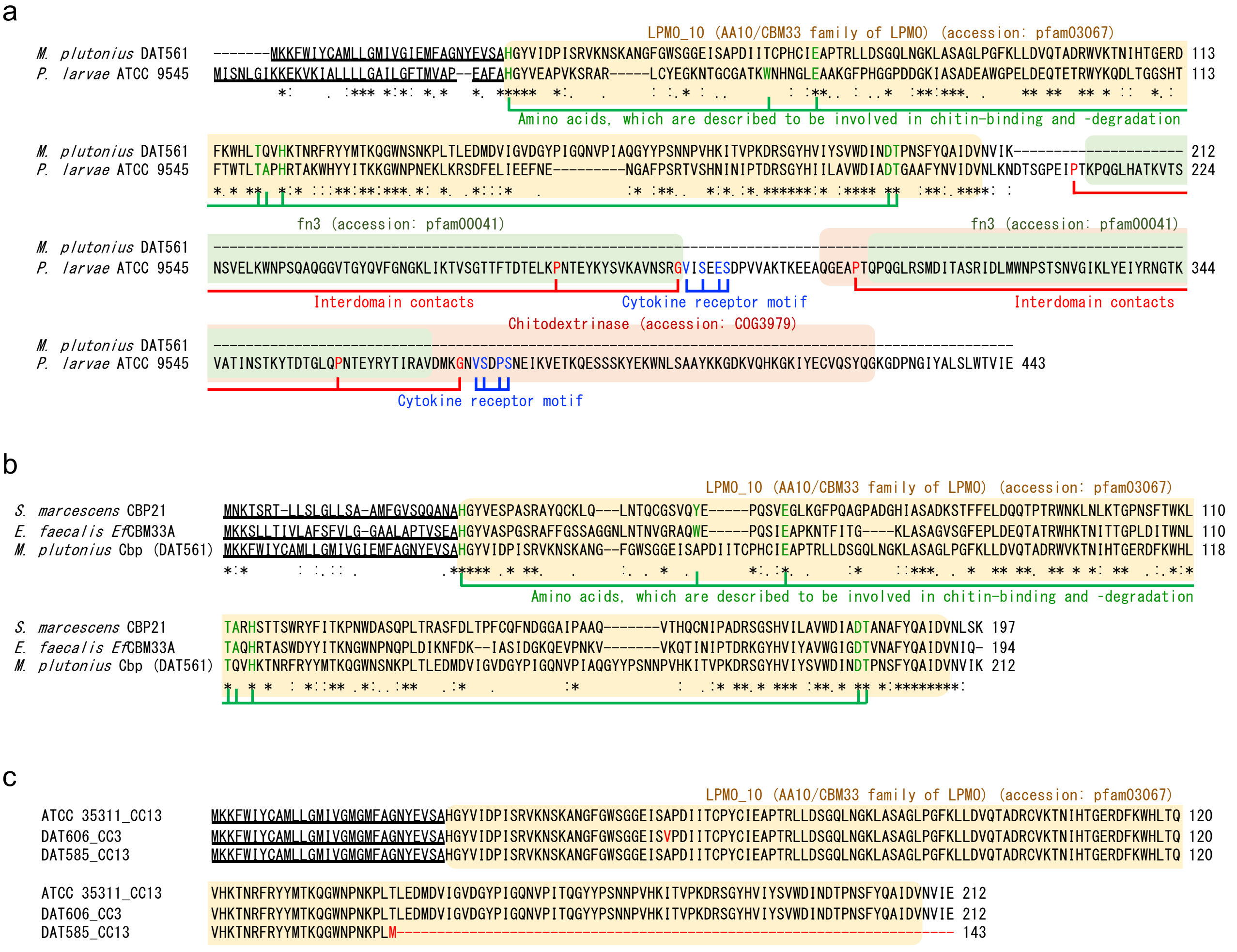

Supplementary Figure S1. Chitin-binding domain-containing proteins (Cbps). (a) Sequence alignment of Cbp of *Melissococcus plutonius* DAT561 (protein id: BBC61089) and *Pl*CBP49 of *Paenibacillus larvae* ATCC 9545 (protein id: AFQ34195). Asterisks below the alignment indicate positions that have a conserved residue. Colons and periods indicate positions that have residues belonging to an amino acid group exhibiting strong and weak similarity, respectively (for further details, see: https://www.ddbj.nig.ac.jp/faq/en/explain-three-symbols-e.html). **(b)** Sequence alignment of Cbp of *M. plutonius* DAT561 (protein id: BBC61089) and two other members of the AA10 family of lytic polysaccharide mono-oxygenases (CBP21 of *Serratia marcescens*, protein id: BAA31569; *Ef*CBM33A of *Enterococcus faecalis*, protein id: AAO80225). Asterisks below the alignment indicate positions that have a single, fully conserved residue. Colons and periods indicate positions that have residues belonging to an amino acid group exhibiting strong and weak similarity, respectively. **(c)** Sequence alignment of Cbps of *M. plutonius* ATCC 35311 (protein id: BAK21424), DAT606 (protein id: BBD16660) and DAT585 (accession no.: AP018524). Red letters represent the amino acid residues different from that of ATCC 35311. Red hyphens indicate the truncated region in DAT585. The alignments were computed using ClustalW (https://clustalw.ddbj.nig.ac.jp/). Conserved domains are highlighted with colored backgrounds. LPMO_10, lytic polysaccharide mono-oxygenase, cellulose-degrading domain, which is annotated as chitin binding domain in *Pl*CBP49; fn3, fibronectin type III domain. Chitodextrinase, the domain that is involved in the carbohydrate transport and metabolism. Underlined sequences are putative signal peptides predicted by SignalP-5.0 (http://www.cbs.dtu.dk/services/SignalP/).

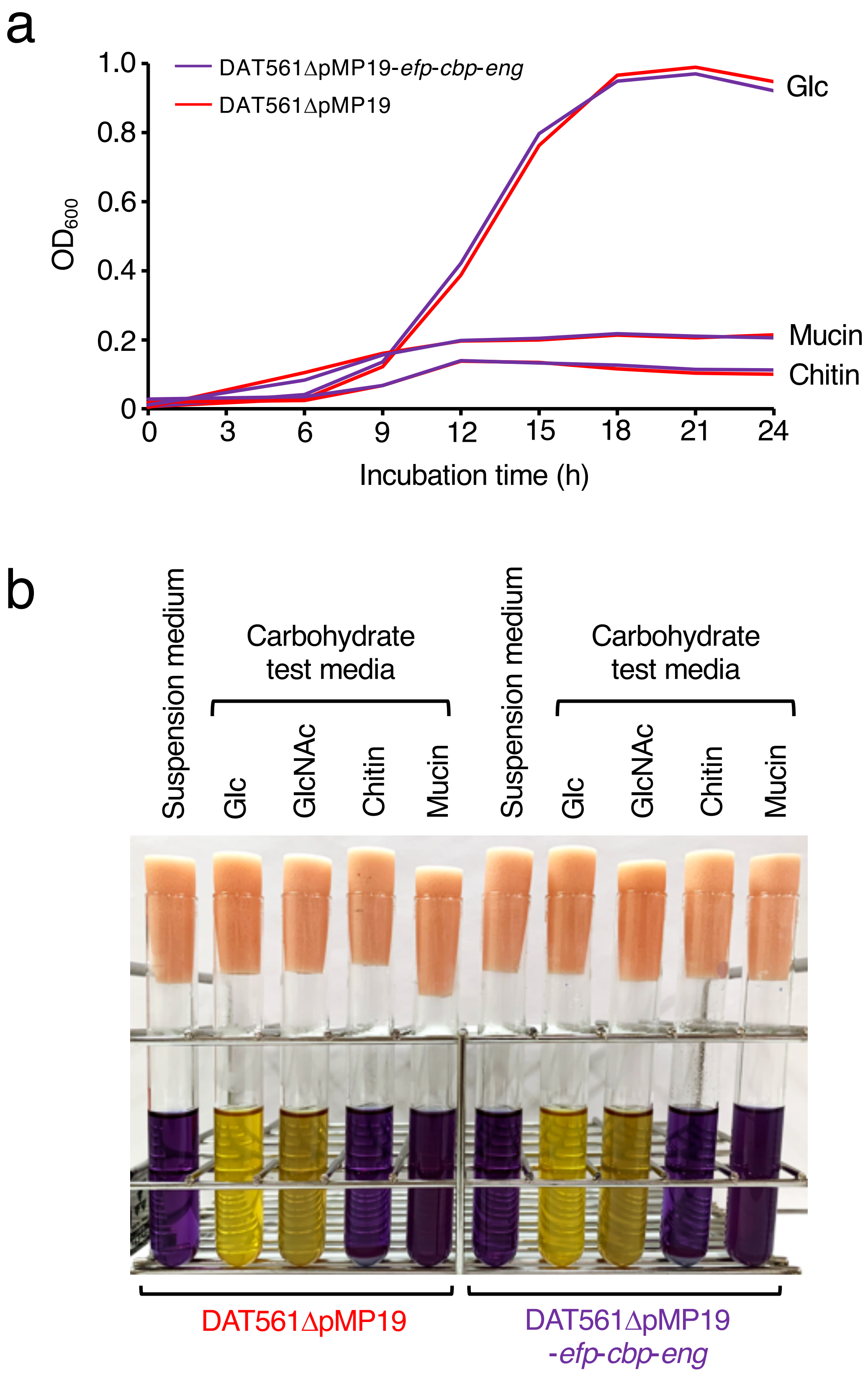

Supplementary Figure S2. Growth of *M. plutonius* DAT561ΔpMP19 and DAT561ΔpMP19-*efp-cbp-eng*. (a) Growth curves in carbohydrate test media supplemented with glucose, chitin and mucin. Means of the optical density at 600 nm (OD_{600}) obtained from five culture tubes are shown. (b) Acid production in carbohydrate test media from supplemented carbon sources. Yellow, acid production positive; Purple, acid production negative. Formulas of suspension and carbohydrate test media are shown in Supplementary Table S6. Glc, glucose; GlcNAc, *N*-acetylglucosamine.
